# To the Editors of the Medical and Physical Journal

**Published:** 1800-07

**Authors:** Edw. Leese

**Affiliations:** East Street, Manchester Square


### *To the Editors of the Medical and Phyſical Journal.*

GENTLEMEN,

AS it has been the opinion of moſt medical men that Small-pox and Meaſles could not exiſt, and go through their different ſtages, at one time, in the ſame perſon, I am induced to tranſmit you the following caſe (that came under my care very lately) to be inſerted in the Medical and Phyſical Journal, if you think it ſufficiently intereſting. I am,

GENTLEMEN,

Very reſpectfully,

*Eaſt Street, Mancheſter Square.* EDW. LEESE.

---

APRIL 19th, 1800. The infant of Mr. W. in Paddington Street, about five months old, at the breaſt, without any appearance of teeth, had been ever ſince its birth a very fine and healthy girl, though it had in the ſkin eight or ten little ſpots ſomething like flea-bites, but ſo very trifling as not to produce in me the leaſt heſitation to inoculate it, it being otherwiſe quite lively, and in good health; I therefore inſerted into the arm ſome variolous matter, recently taken from a healthy girl that had the diſeaſe by inoculation.

On the morning after the inoculation, a powder, compoſed of calom. ppt. and p. rhei, was given; and afterwards, for a few days, pulv. antim. and calom. in very ſmall doſes, every night and morning.

On the 4th day it was evident the virus had began to act in the arm, the little ſpots before obſerved had diſappeared, and the child was in its uſual ſtate of health.

About the 9th day ſome little ſpecks appeared in the ſkin, which it was ſuppoſed would become variolous puſtules, but they diſappeared, though about ten or twelve in the arm, around the inoculated part, continued to increaſe, and advance to ſuppuration.

10th day, Much as yeſterday, but the child rather feveriſh and uneaſy.

11th day, Uneaſy as yeſterday, with ſome little fever; a very few ſpots about the *body*, that promiſed to be variolous puſtules.

12th day, Rather more fever, puſtules more advanced, bowels open; ordered a ſaline febrifuge mixture; ſeemed alſo a little heavy, and ſtuffed at the noſe. The weather being tolerably lerably fine, it had been carried out almoſt every day; was therefore ſuppoſed to have caught a ſlight cold.

13th day, The variolous puſtules advancing, the heat of the ſkin rather leſs; but was ſomewhat ſurprized to find it filled in every part with an eruption exactly reſembling meaſles, accompanied alſo with much redneſs of the eye-lids, and diſcharge from the eyes; the breathing a little impeded from the ſtoppage in the noſe. As I ſuppoſed the variolous and morbillous eruptions could not appear together, but that one would be retarded till the other had taken its courſe, I was in ſome doubt whether to pronounce it meaſles or not.

14th day, The variolous puſtules increaſing, and filled with ſome matter; a good deal of inflammation in the arm, and the puſtules there very prominent, though not very large; the morbillous eruption general, and very diſtinctly marked, with watering of the eyes, ſneezing, cough, and ſlight oppreſſion in reſpiration. I took a medical friend with me, who, like myſelf, had never ſeen ſuch a caſe, and he perfectly agreed with me that there was not the leaſt doubt but the eruption was the meaſles.

15th day, Leſs ill than yeſterday, meaſles diſappearing, ſmall-pox advancing, cough and ſneezing leſs frequent than yeſterday.

16th day, Called, but the child was carried out; the mother informed me it was better than yeſterday; the meaſles nearly gone.

17th day, Meaſles nearly or quite gone, the variolous puſtules dying away alſo; they were ſmall, and contained but little matter; the inoculated part drying, and black in the centre.

18th day, The meaſles entirely diſappeared, and the ſmall-pox going very faſt. From this time the child continued to be in perfect health.

---

